# Direct cord implantation in brachial plexus avulsions: revised technique using a single stage combined anterior (first) posterior (second) approach and end-to-side side-to-side grafting neurorrhaphy

**DOI:** 10.1186/1749-7221-4-8

**Published:** 2009-06-19

**Authors:** Sherif M Amr, Ahmad M Essam, Amr MS Abdel-Meguid, Ahmad M Kholeif, Ashraf N Moharram, Rashed ER El-Sadek

**Affiliations:** 1 The Department of Orthopaedics and Traumatology, Cairo University, Cairo, Egypt; 2 Hand and Microsurgery Service, Al-Helal Hospital, Cairo, Egypt; 3 Department of Orthopaedics and Traumatology, Beni-Suef Faculty of Medicine, Beni-Suef, Egypt; 4 The Department of Orthopaedics and Traumatology, Al-Azhar University, Cairo, Egypt

## Abstract

**Background:**

The superiority of a single stage combined anterior (first) posterior (second) approach and end-to-side side-to-side grafting neurorrhaphy in direct cord implantation was investigated as to providing adequate exposure to both the cervical cord and the brachial plexus, as to causing less tissue damage and as to being more extensible than current surgical approaches.

**Methods:**

The front and back of the neck, the front and back of the chest up to the midline and the whole affected upper limb were sterilized while the patient was in the lateral position; the patient was next turned into the supine position, the plexus explored anteriorly and the grafts were placed; the patient was then turned again into the lateral position, and a posterior cervical laminectomy was done. The grafts were retrieved posteriorly and side grafted to the anterior cord. Using this approach, 5 patients suffering from complete traumatic brachial plexus palsy, 4 adults and 1 obstetric case were operated upon and followed up for 2 years. 2 were C5,6 ruptures and C7,8T1 avulsions. 3 were C5,6,7,8T1 avulsions. C5,6 ruptures were grafted and all avulsions were cord implanted.

**Results:**

Surgery in complete avulsions led to Grade 4 improvement in shoulder abduction/flexion and elbow flexion. Cocontractions occurred between the lateral deltoid and biceps on active shoulder abduction. No cocontractions occurred after surgery in C5,6 ruptures and C7,8T1 avulsions, muscle power improvement extended into the forearm and hand; pain disappeared.

**Limitations include:**

spontaneous recovery despite MRI appearance of avulsions, fallacies in determining intraoperative avulsions (wrong diagnosis, wrong level); small sample size; no controls rule out superiority of this technique versus other direct cord reimplantation techniques or other neurotization procedures; intra- and interobserver variability in testing muscle power and cocontractions.

**Conclusion:**

Through providing proper exposure to the brachial plexus and to the cervical cord, the single stage combined anterior (first) and posterior (second) approach might stimulate brachial plexus surgeons to go more for direct cord implantation. In this study, it allowed for placing side grafts along an extensive donor recipient area by end-to-side, side-to-side grafting neurorrhaphy and thus improved results.

**Level of evidence:**

Level IV, prospective case series.

## Background

Although intradural exploration of the brachial plexus had been reported in 1911 [1] and surgical repair of an intraspinal plexus lesion had been performed in 1979 [2], directly implanting avulsed roots into the spinal cord stimulated the interest of surgeons for a short period of time before falling into disrepute. The fact is, following avulsion of the nerve roots off the spinal cord, successful recovery of function depends on several factors [3,4]. Firstly, new nerve fibers have to grow along a trajectory consisting of central nervous system growth-inhibitory tissue in the spinal cord as well as peripheral nervous system growth-promoting tissue in nerves. Secondly, local segmental spinal cord circuits have to be reestablished. Thirdly, a large proportion of motoneurons die shortly after the injury. Schwann cells are one of the major sources of neurotrophic factors, particularly those relating to the survival of motoneurons, such as ciliary neurotrophic factor (CNTF) and brain-derived neurotrophic factor. In root avulsions, the loss of peripheral connection leads to loss of this local source of trophic support and subsequent apoptosis, but ischaemic cell death might also occur.

Nevertheless, regrowth of motoneuron axons into neighboring ventral nerve roots after lesions was proven in the pioneering studies by Ramon y Cajal [5] and later confirmed in several other experimental studies [6,7]. The scar tissue within the spinal cord was shown to be conducive to regeneration [3]. Clinically, interest in direct cord implantation was rekindled in 1995, when Carlstedt et al. [8] described the implantation of a ventral nerve root and nerve grafts into the spinal cord in a patient with brachial plexus avulsion injury. Results of surgery were reported in several other studies [3,8-11].

Although the technique is expected to solve the problem of multiple root avulsions, it has found only limited application among brachial plexus surgeons. The fact is, current surgical approaches for direct cord implantation provide only limited exposure either to the brachial plexus or to the cervical cord, cause much tissue damage and lack extensibility. Using a single stage combined anterior (first) posterior (second) approach, we describe a technique that provides adequate exposure to the brachial plexus and to the cervical cord, causes minimal tissue damage, is extensible and allows for ample placement of nerve grafts along the cervical cord and roots, trunks and cords of the brachial plexus.

## Methods

### Patients

5 patients suffering from complete traumatic brachial plexus palsy, 4 adults and 1 obstetric case, were operated upon from 2005 up to 2006 and followed up for 2 years. At the time of surgery, the ages of the adult subjects ranged from 27 up to 45 years with a median of 37 years; all were male. 2 adult patients suffered from a (C5,6 rupture C7,8T1 avulsion), 2 were (C5,6,7,8T1 avulsions); all were operated upon within 1 year after injury. The obstetric case was a (C5,6,7,8T1 avulsion) and was operated upon at 1 year of age. The demographic data, clinical and operative findings and operative procedures are presented in Table 1.

**Table 1:** The demographic data of the patients, lesion types, operative procedures, preoperative cocontractions and deformities and the pre- and postoperative evaluation scores

Pt	Age (yrs) sex	Type of injury	Time of surgery after injury (mths)	Procedure	Nerve grafts	Associated injuries	Deformities	Shoulder score Narakas (N), Gilbert (G)	Elbow score Waikakul (W). Gilbert (G)	Hand score Raimondi (R)
1	27 M	C5,6,7,8T1 avulsion lt. brachial plexus; retraction of the brachial plexus to the deltopectoral groove	8	Direct cord implantation	Both surals	Retroclaclavicular CSF sac; neglected rupture of subclavian artery; delayed union of fracture of the lt. humerus	Volkmann's ischaemic contracture	Ngood	Wgood	R0

2	40 M	C5,6,7,8T1 avulsion rt. brachial plexus; retraction of the brachial plexus to the deltopectoral groove	12	Direct cord implantation	Both surals	grafted subclavian artery	-	Ngood	Wgood	R0

3	45 M	C5,6 rupture C7,8T1 avulsion rt. brachial plexus; retraction of the brachial plexus to the outer border of scalenus anterior muscle	8	C5,6 grafting to superior trunk, C7,8T1: direct cord implantation	Medial cutaneous nerve of forearm, superficial radial nerve, supraclavicular nerves	-	-	Nexcellent	Wexcellent	R3

4	30 M	C5,6 rupture C7,8T1 avulsion lt. brachial plexus; retraction of the brachial plexus to the clavicle	8	C5,6 grafting to superior trunk, C7,8T1: direct cord implantation	Medial cutaneous nerve of forearm, superficial radial nerve, supraclavicular nerves	-	-	Nexcellent	Wexcellent	R3

5	1 M	C5,6,7,8T1 avulsion rt. brachial plexus; retraction of the brachial plexus to the clavicle; obstetric palsy	12	Direct cord implantation	Medial cutaneous nerve of forearm, superficial radial nerve			Ngood, G3	Ggood	R4

### Patient evaluation

All patients were evaluated pre- and postoperatively (every 2 months) for deformities, muscle function and cocontractions. To limit intraobserver and interobserver variability, testing for deformities, muscle function and cocontractions was recorded by digital photography on both normal and healthy sides. The normal side was recorded to ensure the patient had complied with the examiner's instructions. Electromyographic studies were performed preoperatively. Although CT cervical myelography is more accurate than magnetic resonance imaging in evaluating root avulsions [12], patients accepted magnetic resonance imaging more readily. Magnetic resonance imaging was reported to have a 81% sensitivity in detecting root avulsions [13]. Thus, root avulsions were evaluated by magnetic resonance imaging and confirmed intraoperatively [14].

### Range of motion and deformities

The range of elbow flexion was measured as the angle formed between the long axis of the arm and the forearm. The range of abduction was recorded by measuring the angle formed between the arm axis and parallel to the spinal cord axis. External rotation was measured with the patient standing with the shoulder fully internally rotated and forearm placed transversally over the abdomen. Any rotation from this position was measured and noted as the range of external rotation [15].

In all adult patients, the shoulders and elbows were flail. The wrist and fingers were stiff in extension in 2 patients, while 1 patient presented with a Volkmann's ischaemic contracture of the forearm and hand (Table 1).

### Muscle function

Muscle function was assessed using the system described in the report of the Nerve Committee of the British Medical Council in 1954 and previously used by other authors [16]. The anterior, middle and posterior deltoid were tested separately [17]. The subscapularis was tested by the lift-off test and the lift-off lag sign [18-20]. The supraspinatus was tested using Jobe's empty can test. The infraspinatus integrity is tested by the external rotation lag (dropping) sign, by Hornblower's sign and by the drop arm sign. These tests were modified to test for muscle power. Although all of the above tests were reliable, the most sensitive test was the drop arm test [18]. Some reports questioned its sensitivity, however [20]. In the current study, when the patient could actively abduct his shoulder, the drop arm sign was used, as it was the most sensitive; otherwise, the other two tests were used. In testing finger flexors and extensors, both elbows and wrists were immobilized on a board.

### Evaluation for cocontractions

Cocontractions were evaluated by asking the patient to abduct the shoulder without actively flexing, internally or externally rotating it and without actively moving the elbow, forearm, wrist or fingers [21]. He was observed if he could abduct the shoulder independently of other movements. The same procedure was repeated for shoulder flexion, elbow flexion and extension, forearm pronation and supination, wrist and finger flexion and extension.

### Functional scoring

Shoulder function was graded using the scale proposed by Narakas [15,21-23] (poor: no abduction movement and feeling of weightlessness in the limb (motor power grade 0); fair: stable shoulder without any subluxation but no active movement (motor power grade 1); good: active abduction of < 60 degrees (motor power grade 3) and active external rotation of < 30 degrees; excellent: active abduction of > 60 degrees (motor power grade 4) and active external rotation of > 30 degrees).

Elbow function was graded using the scale proposed by Waikakul et al. [15,24] (excellent: ability to lift 2 kg weight from 0 to 90 degrees of elbow flexion more than 30 times successively; good: ability to lift 2 kg weight from 0 to 90 degrees of elbow flexion, but less than 30 repetitions successively; fair: motor power more than grade 3 but unable to lift a 2 kg weight; poor: motor power less than grade 3).

The paediatric case was evaluated using the Gilbert shoulder and elbow scales [21,22] (shoulder scale: Grade 0: completely paralysed shoulder or fixed deformity; Grade 1: abduction = 45 degrees, no active external rotation; Grade 2: abduction < 90 degrees, bioactive external rotation; Grade 3: abduction = 90 degrees, active external rotation < 30 degrees; Grade 4: abduction < 120 degrees, active external rotation 10–30 degrees; Grade 5: abduction > 120 degrees, active external rotation 30–60 degrees; Grade 6: abduction > 150 degrees, active external rotation > 60 degrees). The Gilbert elbow scale included the following items: flexion (1: no or minimal muscle contraction, 2: incomplete flexion, 3: complete flexion); extension (0: no extension; 1: weak extension; 2: good extension); flexion deformity (extension deficit) (0: 0–30 degrees, -1:30–50 degrees, -2:> 50 degrees). Evaluation was as follows: 4–5 points: good regeneration; 2–3 points: moderate regeneration; 0–1 points: bad regeneration.

The Raimondi hand evaluation scale [21,22] comprised the following grades: Grade 0: complete paralysis or minimal useless finger flexion; Grade 1: useless thumb function, no or minimal sensation, limitation of active long finger flexors; no active wrist or finger extension, key-grip of the thumb; Grade 2: active wrist extension; passive long finger flexors (tenodesis effect); Grade 3: passive key-grip of the thumb (through active thumb pronation), complete wrist and finger flexion, mobile thumb with partial abduction, opposition, intrinsic balance, no active supination; Grade 4: complete wrist and finger flexion, active wrist extension, no or minimal finger extension, good thumb opposition with active intrinsic muscles (ulnar nerve), partial pronation and supination; Grade 5: as in Grade 4 in addition to active long finger extensors, almost complete thumb pronation and supination.

#### Pain

In adults, the presence or absence of pain and its degree were assessed on a visual analogue scale from 1 to 5.

### Operative procedure

#### Draping of the patient

**Figure 1:**
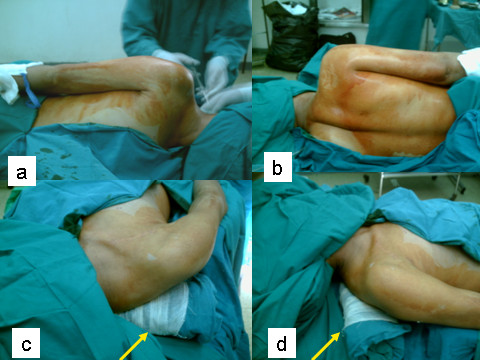
**a-d – The patient is sterilized and draped in the lateral position**. The sterilization area includes: the front and back of the neck, the front and back of the chest up to the midline and the whole affected upper limb. Next the patient is turned into the usual supine position for anterior exploration of the brachial plexus. To help extend the shoulders, a sterile pad is placed posteriorly between them (yellow arrow). A head pad supports the head. The head is turned to the contralateral side.

The patient was prepared and draped in the lateral position, the affected side up. A pad helped elevate the head. The sterilization area included: the front and back of the neck, the front and back of the chest up to the midline and the whole affected upper limb (Figs. 1a and 1b). Both lower limbs served as donor sites for sural nerve grafts and were sterilized.

#### Turning the patient into the supine position

Next the patient was turned into the usual supine position for anterior exploration of the brachial plexus. To help extend the shoulders, a sterile pad was placed posteriorly between them. A head pad supported the head. The head was turned to the contralateral side (Figs. 1c and 1d).

#### Conventional anterior exploration of the brachial plexus

After that, the brachial plexus was explored anteriorly as usual. We preferred to explore it through a transverse supraclavicular incision with a deltopectoral extension, yet without clavicular osteotomy [14]. After cutting the clavicular head of the sternomastoid and the insertion of scalenus anterior muscle medially, and the clavicular and part of acromial insertion of the trapezius muscle laterally [25,26], exploration of the brachial plexus proceeded as described elsewhere [14,27-29].

**Figure 2:**
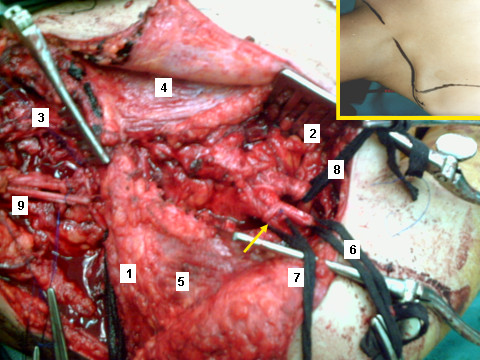
**In C5,6,7, 8T1 avulsions and using the principle of closed loop of end-to-side side-to-side grafting neurorrhaphy, one nerve graft is looped through the superior and middle trunks and lateral and posterior cords and another nerve graft is looped through the inferior trunk, medial cord and medial root of median nerve (1: clavicle; 2: deltopectoral groove; 3: supraclavicular area; 4: pectoralis major; 5: deltoid; 6: lateral cord; 7: posterior cord; 8: medial cord; 9: grafts having been passed beneath the clavicle into the supraclavicular area; arrow: grafts looped into the cords)**. The inset shows the position of the patient and the incision line.

**Figure 3:**
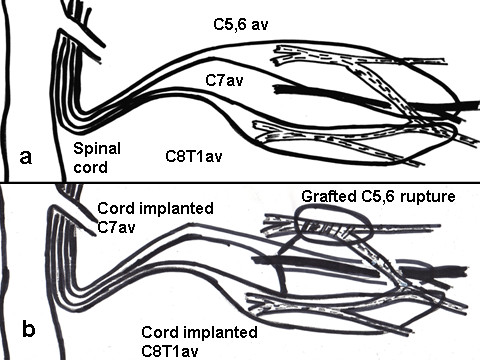
**a and b – Schematic drawing showing the importance of placing grafts in an end(graft)-to-side(cord) and side(graft)-to-side(cord) fashion over an extensive area along the anterior cord to increase the chances of side neurotization**. It also shows the technique of closed loop grafting as explained in Fig. 2. In (C5,6,7, 8T1 avulsions), one nerve graft is looped through the superior and middle trunks and lateral and posterior cords and another nerve graft is looped through the inferior trunk, medial cord and medial root of median nerve. In (C5,6 ruptures C7,8 T1 avulsions) the closed loop technique of end-to-side side-to-side grafting neurorrhaphy is used to graft the ruptured C5,6 roots to the superior trunk of the brachial plexus. Next, one nerve graft is looped through the middle trunk and posterior cord and another is looped through the inferior trunk, medial cord and medial root of median nerve.

In Cases 1, 2, 5 (C5,6,7, 8T1 avulsions), aiming at direct cord implantation and using the principle of closed loop of end-to-side side-to-side grafting neurorrhaphy [30], one nerve graft was looped through the superior and middle trunks and lateral and posterior cords and another nerve graft was looped through the inferior trunk, medial cord and medial root of median nerve. In Cases 3 and 4 (C5,6 ruptures C7,8 T1 avulsions) the closed loop technique of end-to-side side-to-side grafting neurorrhaphy [30] was used to graft the ruptured C5,6 roots to the superior trunk of the brachial plexus. Next, aiming at direct cord implantation, one nerve graft was looped through the middle trunk and posterior cord and another was looped through the inferior trunk, medial cord and medial root of median nerve (Figs. 2 and 3a, b).

#### Turning the patient into the lateral position again

The sterile pad between the shoulders was removed and the patient was turned again into the lateral position. Contrary to conventional fascicular epiperineurial neurorrhaphy, closed looping provided a stable graft recipient junction, which allowed turning the patient again into the lateral position to approach the cervical cord posteriorly

#### Exposing the cervical cord through a conventional posterior cervical laminectomy

Through a midline skin incision extending from the occiput to the posterior process of T1, and using the midline intermuscular plane of the posterior neck muscles, the cervical laminae were exposed. A cervical laminectomy was carried out.

#### Retrieving the nerve graft loops into the posterior laminectomy

**Figure 4:**
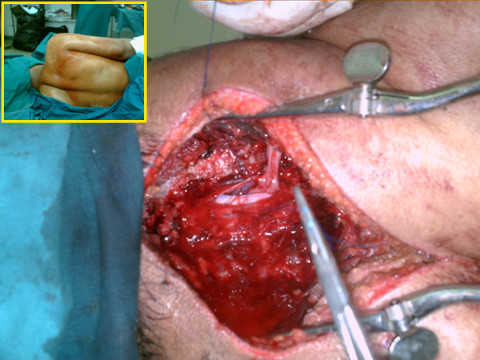
**A posterior cervical laminectomy, while the patient is in the lateral position; the right shoulder is in the upper right corner; the head is on the left**. The dura has been incised. The grafts have been passed through the dural incision and placed in an end(graft)-to-side(cord) and side(graft)-to-side(cord) fashion for about 4 cms along the anterior cord close to the midline sulcus in a subpial plane. They are held in place by placing them anterior to C4 intradural cervical nerve root proximally and T1 intradural nerve root distally. In 5 minutes, they adhere to the cord. The dura is closed using 3/0 prolene continuous sutures. The inset shows the position of the patient.

Through the posterior incision and using a submuscular plane, a right-angled dissection forceps was inserted along the posterior aspect of C7 transvserse process, and entered into the anterior incision. It was used to hold the proximal free ends of the graft loops and pull them gently into the posterior laminectomy incision (Fig. 4)

#### Opening the dura

The dura was next opened posteriorly using a 11-scalpel blade. Its edges were kept open by means of 3/0 prolene sutures. A dural dissector was used to cut the dentate ligaments and clear the pia mater off the anterior cord from C4 up to C7. The avulsed roots were explored intradurally. Thus extending the laminectomy by a partial facetectomy on the injured side of the brachial plexus to fully expose every root and provide adequate working space for the subsequent repair was avoided lest the spine should be destabilized.

#### Inserting the proximal ends of the graft into the anterior cord

**Figure 5:**
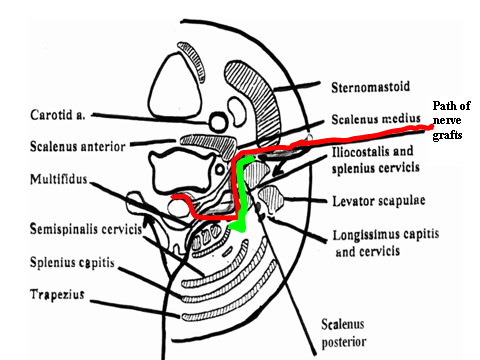
**The combined approach for direct cord implantation**. Conventional anterior dissection (anterior bifurcated black arrow) provides access to the roots, trunks and cords of the brachial plexus. Approaching the cervical cord through a conventional laminectomy (posterior bifurcated black arrow) provides adequate exposure and allows for lateral retraction of the paraspinal musculature, thus preserving their segmental nerve and vascular supply. Through the posterior incision and using a submuscular plane, a right-angled dissection forceps is inserted along the posterior aspect of C7 transvserse process, and entered into the anterior incision (bright green line). It is used to hold the proximal free ends of the graft loops and pull them gently into the posterior laminectomy incision. The red line shows the path of the nerve grafts.

The grafts were passed through the dural incision and placed in an end(graft)-to-side(cord) and side(graft)-to-side(cord) fashion for about 4 cms along the anterior cord close to the midline sulcus in a subpial plane (Figs 3 and 5). They were held in place by placing them anterior to C4 intradural cervical nerve root proximally and T1 intradural nerve root distally. In 5 minutes, they adhered to the cord. The dura was closed using 3/0 prolene continuous sutures.

#### Wound closure

The wound was closed in layers.

#### Postoperative immobilization

**Figure 6:**
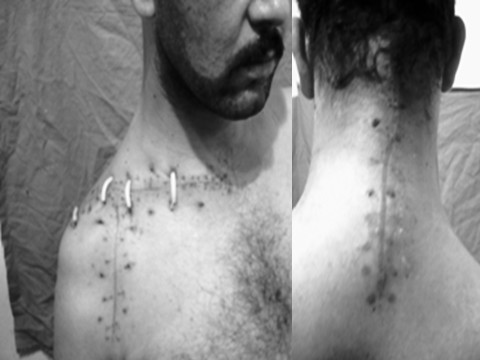
**A postoperative picture illustrating the incision lines of the combined approach**.

The patient's neck was immobilized postoperatively in a soft collar for 6 weeks. Figure 6 shows a postoperative picture illustrating the incision lines of the combined approach

### Donor nerves

Both sural nerves, the superficial radial nerve and the medial cutaneous nerve of the forearm and the supraclavicular nerves served as nerve grafts.

## Results

### Technical advantages

#### Anterior exposure

As both supraclavicular and infraclavicular parts of the brachial plexus were explored, the extent of the injury could be estimated. Cases 1, 2 and 5 were C5,67,8T1 avulsions; the brachial plexus was retracted to the deltopectoral groove in Cases 1 and 2 and to the clavicle in Case 5. Cases 3 and 4 were C5,6 ruptures C7,8T1 avulsions

The brachial plexus was retracted to the outer border of scalenus anterior in Case 3 and to the clavicle in Case 5.

#### Posterior exposure

As the whole cervical cord was explored adequately, the extent of root avulsions could be determined accurately. It was used to confirm the findings obtained from MRI and anterior exposure.

### Complications of surgery

None of our patients lost neurologic function, had CSF leak or developed myelitis as a result of cord manipulation. None suffered from cervical pain or developed cervical instability as a result of the laminectomy. The paediatric case complained of mild hyperextension of the neck as a result of contracture of the posterior laminectomy scar.

### Motor power

Improvements in motor power are shown in Table 2 [see additional file 1].

#### Motor power in C5,6 ruptures C7,8T1 avulsions

In Cases 3 and 4, the biceps and anterior deltoid improved from Grade0 to Grade5; the lateral and posterior deltoid, the supra- and infraspinatus, the subscapularis, pectoral and clavicular heads of pectoralis major, latissimus dorsi, triceps improved from Grade 0 to Grade 4. The pronator teres, extensor carpi ulnaris, flexor digitorum profundus and flexor pollicis longus improved from Grade 0 to Grade 3. The flexor digitorum superficialis improved from Grade 0 to Grade 2.

#### Motor power in C5,6,7,8T1 avulsions

In Cases 1 and 2, the biceps and anterior, lateral and posterior deltoid, the supraspinatus, the subscapularis, pectoral and clavicular heads of pectoralis major, latissimus dorsi, improved from Grade 0 to Grade 4. The infraspinatus, triceps and pronator teres improved from Grade 0 to Grade 3.

In Case 5, the biceps and anterior deltoid improved from Grade0 to Grade5; the lateral and posterior deltoid, the supraspinatus, the subscapularis, pectoral and clavicular heads of pectoralis major, latissimus dorsi, triceps improved from Grade 0 to Grade 4. The infraspinatus, pronator teres, extensor carpi ulnaris, extensor carpi radialis longus and brevis, flexor carpi ulnaris, flexor carpi radialis, thumb and finger extensors, flexor digitorum profundus and superficialis, flexor pollicis longus improved from Grade 0 to Grade 3. The intrinsic muscles of the hand improved from Grade 0 to Grade 2.

### Cocontractions

#### C5,6 ruptures C7,8T1 avulsions

No cocontractions were recorded in Cases 3 and 4.

#### C5,6,7,8T1 avulsions

In Cases 1 and 2 cocontractions occurred between the lateral deltoid and biceps on active shoulder abduction.

In Case 5, cocontractions occurred between the lateral deltoid, biceps and finger extensors on active shoulder abduction.

### Functional Score

#### Shoulder score

- C5,6 ruptures C7,8T1 avulsions:

Cases 3 and 4 achieved a Narakas score of excellent

- C5,6,7,8T1 avulsions:

Because of weak shoulder external rotation, Cases 1, 2, and 5 achieved a Narakas score of good. Case 5 achieved also a Grade3 Gilbert score.

#### Elbow score

- C5,6 ruptures C7,8T1 avulsions:

Cases 3 and 4 achieved a Waikakul score of excellent

- C5,6,7,8T1 avulsions:

Cases 1 and 2 achieved a Waikakul score of good.

Case 5 achieved a Gilbert score of good.

#### Hand score

- C5,6 ruptures C7,8T1 avulsions:

Cases 3 and 4 improved from a Raimondi score of 0 to a score of 3.

- C5,6,7,8T1 avulsions:

Cases 1 and 2 remained with a Raimondi score of 0.

Case 5 improved from a Raimondi score of 0 to a score of 4.

### Pain

In adult total avulsions (Cases 1 and 2), pain persisted and had a grade of 4. In C5,6 ruptures C7,8T1 avulsions, pain disappeared, but patients complained of a sensation of tingling on combined shoulder flexion and elbow extension.

## Discussion

Six issues have to be addressed in this work: 1. approaching the brachial plexus surgically for purpose of cord implantation; 2. side-to-side end-to-side grafting neurorrhaphy between the recipient brachial plexus and the distal aspect of the nerve graft conduits; 3. side-to-side end-to-side grafting neurorrhaphy between the donor anterior aspect of the cervical cord and the proximal ends of the nerve graft conduits; 4. the role of direct cord implantation in complete avulsions; 5. the role of direct cord implantation in incomplete avulsions; 6. shortcomings of the technique and future directions; 7. limitations of the study.

### Approaching the brachial plexus surgically for purpose of cord implantation

Approaching the brachial plexus surgically for purpose of cord implantation is the first issue we have to address.

Conventional anterior approaches to the brachial plexus [14,27-29] afford good exposure to the anterior structures. Yet, a facetectomy, foraminotomy or hemilaminectomy cannot be performed through them. Juergens-Becker et al. [31] performed a diagnostic foraminotomy through a posterior approach as a first stage. At a second stage, anterior exploration of the brachial plexus was carried out.

**Figure 7:**
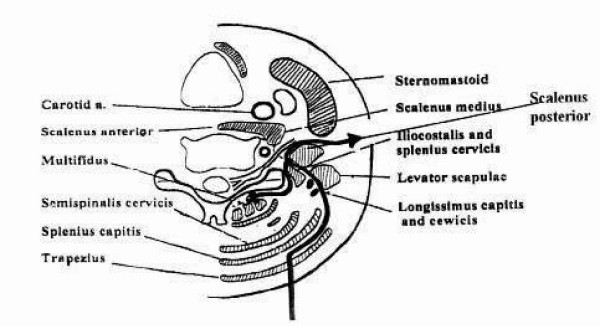
**In the posterior subscapular approach, the trapezius muscle rhomboideus minor and major muscles are divided longitudinally away from their nerve supply**. The anterior plane of dissection is developed by disinserting the levator scapulae, the posterior and middle scalene muscles and retracting them anteriorly and superiorly (anterior black arrow); this provides only limited exposure to the brachial plexus. The posterior plane of dissection is developed by medial retraction of the paraspinal muscles (posterior black arrow). The latter muscles are too bulky to be retracted medially adequately. Besides, medial retraction damages their nerve and vascular supply.

Using the posterior subscapular approach [32,33] (Fig. 7), Carlstedt [8] was able to approach the laminae, facet joints and avulsed root stumps present within the spinal canal. He was not able to reach those roots avulsed out of the spinal canal and migrated distally [8]. In the posterior subscapular approach [32], the trapezius muscle was divided longitudinally away from its nerve supply, the levator scapulae, the rhomboideus minor and major muscles were exposed and divided away from the edge of the scapula. Thus, the posterior chest wall was exposed. The ribs were then palpated, the first rib was located and removed extraperiosteally, from the costotransverse articulation posteriorly to the costoclavicular ligament anteriorly. The posterior and middle scalene muscles were released from their origin from the transverse spinous processes. After removal of these muscles superiorly, the roots of spinal nerves and the trunks of the brachial plexus were exposed and traced back to the spine. Some elevation and retraction of the paraspinous muscle mass exposed the lateral posterior spine overlying the intraforminal course of the spinal nerves.

From that description, it is evident, that this approach affords little exposure to the anterior structures, namely the trunks, cords and divisions of the brachial plexus, the subclavian vessels and their branches. This approach affords also limited exposure to the posterior structures, namely the cord and intradural nerve roots. Furthermore, it lacks extensibility. As it does not pass through proper intermuscular-internervous planes, it produces damage to the muscles and their vascular supply.

**Figure 8:**
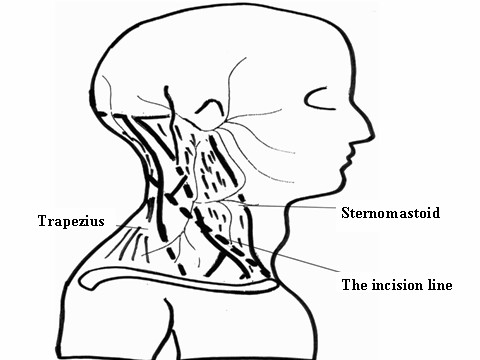
**In the extreme lateral approach, the skin incision extends from the sternoclavicular joint and is continued in the posterior triangle of the neck in a lateral and cranial direction, toward the spinous processes of C4-5 (dashed line); thus there is but limited access to the extraspinal brachial plexus**.

**Figure 9:**
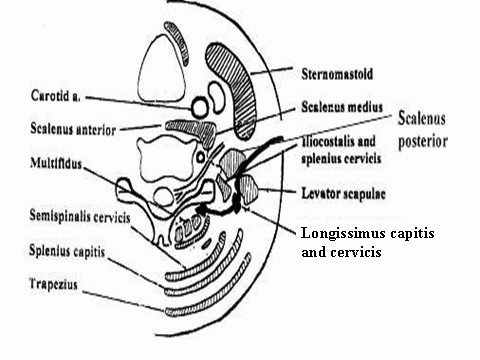
**The extreme lateral approach**. To approach the cord posteriorly, the transverse processes of C4-7 are approached through a connective tissue plane between the levator scapula and the posterior and medial scalenus muscles. The longissimus muscle has to be split longitudinally to approach the posterior tubercles of the transverse processes (black arrow). As mentioned before, the paraspinal muscles are too bulky to be retracted medially adequately. Besides, medial retraction damages their nerve and vascular supply.

The lateral approaches to the crevical spine provide only a partial answer to this problem [34-36]. To expose the upper cervical spine, Crockard et al. [37] placed the patient in the lateral position, entered the cervical spine posterior to the sternomastoid, the levator scapulae and splenius cervicis muscles. Later on, Carlstedt used the extreme-lateral approach [3] (Figs. 8 and 9) to access both the intra-and the extraspinal parts of the plexus. The patient was placed in a straight lateral position. The head was held in a Mayfield clamp with the neck slightly flexed laterally to the opposite side. A skin incision was made in the region of the sternoclavicular joint and continued in the posterior triangle of the neck in a lateral and cranial direction, toward the spinous processes of C4-5. The accessory nerve was identified and protected as it emerged from the dorsal aspect of the cranial part of the sternocleidomastoid muscle. The extraspinal portion of the plexus was next dissected. The transverse processes of C4-7 were approached through a connective tissue plane between the levator scapula and the posterior and medial scalenus muscles. The longissimus muscle had to be split longitudinally to approach the posterior tubercles of the transverse processes. The paravertebral muscles were dissected free from the hemilaminae and pushed dorsomedially. After performing a hemilaminectomy, the dura mater was incised longitudinally.

Thus, lateral approaches to the spine not only suffer from the same disadvantages described previously, but they also afford little exposure to the cord and intradural nerve roots, thus limiting the area of side neurotization to the cord.

For our part, we described an extended anterior and posterior approach to the brachial plexus [25]. The brachial plexus was exposed through a standard L-shaped incision with a deltopectoral extension as described by other authors [14,27-29].

**Figure 10:**
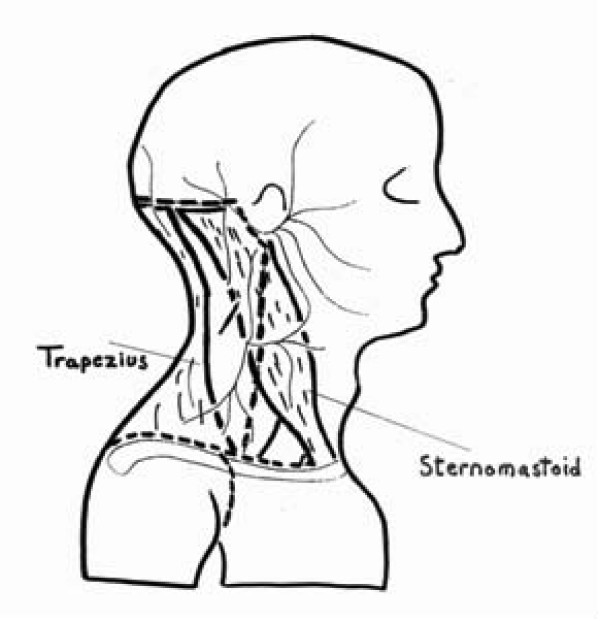
**In the extended approach, the brachial plexus is exposed through a standard L-shaped incision with a deltopectoral extension (dashed line)**. Extending the horizontal limb of the L-incision posteriorly, the trapezius muscle is disinserted from the clavicle and acromion process. Extending the vertical limb of the L-incision horizontally along the superior nuchal line, the origin of the trapezius muscle from the superior nuchal line and the external occipital protuberance is cut and the spinal accessory nerve followed to its motor point into the trapezius muscle. Next, the muscle itself is reflected posteriorly to expose the levator scapulae muscle anteriorly and the splenius capitis muscle posteriorly. This done, the splenius capitis and semispinalis capitis musles is disinserted from the occiput and reflected posteriorly as well.

**Figure 11:**
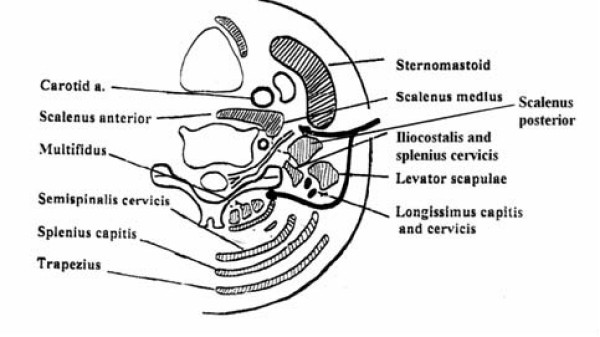
**To expose the posterior structures in the extended approach, the plane posterior to the following muscles is located: the levator scapulae, the iliocostalis cevicis and the longissimus capitis and cervicis**. Anterior retraction of these muscles and medial retraction of the semispinalis cervicis and multifidus muscles allows exposure of the facet joints and performing a facetectomy (posterior black arrow).

Extending the horizontal limb of the L-incision posteriorly, the trapezius muscle was disinserted from the clavicle and acromion process. Extending the vertical limb of the L-incision horizontally along the superior nuchal line, the origin of the the trapezius muscle from the superior nuchal line and the external occipital protuberance was cut and the spinal accessory nerve was followed to its motor point into the trapezius muscle. Next, the muscle itself was reflected posteriorly to expose the levator scapulae muscle anteriorly and the splenius capitis muscle posteriorly. This done, the splenius capitis and semispinalis capitis musles were disinserted from the occiput and reflected posteriorly as well. The plane posterior to the following muscles was located: the levator scapulae, the iliocostalis cevicis and the longissimus capitis and cervicis. Anterior retraction of these muscles and medial retraction of the semispinalis cervicis and multifidus muscles allowed us to expose the facet joints and perform a facetectomy (Figs. 10 and 11).

The problems we met with in this approach were sloughing of the fat pad covering the brachial plexus due to its extensive dissection; sloughing of the tip of the skin flap at the medial end of the lower horizontal skin incision; bleeding from the vertebral artery; bleeding from the cranial vessels, which lay between the semispinalis capitis and the semispinalis cervicis; CSF leakage from meningoceles.

A two stage combined posterior (first) anterior (second) approach was introduced [38] that provided adequate exposure to the brachial plexus and to the cervical cord. These advantages were undermined by operating in two stages. In the first stage, one end of the harvested sural nerve graft was implanted into the ventral lateral aspect of the spinal cord; the other end was identified with a small segment of Foley catheter and radioopaque marker hemoclips and inserted carefully into the paraspinal muscles toward the anterior suprascapular region. Several days later, and through an anterior supraclaviclar approach, the Foley catheter segment was dug out with or without fluoroscopic guidance, removed and the nerve graft anastomosed to the trunk level of the brachial plexus. Thus, extensive tissue damage might occur by having to identify the sural nerve grafts through the paraspinal muscles several days later. Also, as the grafts were invariably inserted into the anterior suprascapular region to be anastomosed several days later to the trunk level of the brachial plexus, no account was taken of the severity of the brachial plexus lesion itself, which might lead to retraction of the avulsed roots up to the deltopectoral or axillary areas (e.g. Cases 1 and 2 in this study), necessitating tailoring grafts to extend to the latter sites. Although Juergens-Becker et al. [31] performed a diagnostic foraminotomy through a posterior approach as a first stage, the presence or absence of root avulsions or ruptures, the degree of retraction of the brachial plexus, the extension of fibrosis and scarring along the brachial plexus are all determinants which can only be properly estimated after anterior (first) exploration of the brachial plexus. Root avulsions could be confirmed after that through a posterior laminectomy. Preoperative investigations to determine root avulsions merely help the surgeon devise the operative technique.

These complications prompted us to devise a single stage combined anterior (first) posterior (second) approach for purpose of direct cord implantation. Both approaches passed through anatomical planes and were extensible. The anterior approach afforded good exposure to the roots, trunks, divisions and cords of the brachial plexus, while the posterior approach provides good exposure to the cervical cord and roots of the brachial plexus. The patient was prepared and draped in the lateral position, the affected side up. A pad helped elevate the head. The sterilization area included: the front and back of the neck, the front and back of the chest up to the midline and the whole affected upper limb. Next the patient was turned into the usual supine position for anterior exploration of the brachial plexus and the brachial plexus explored anteriorly as usual; ruptures were grafted. Nerve grafts were looped into the recipient avulsed nerves. This done, the patient was turned again into the lateral position and the cervical cord exposed just like a conventional cervical laminectomy. Through the posterior incision and using a submuscular plane, a right-angled dissection forceps was inserted along the posterior aspect of C7 transvserse process, and entered into the anterior incision. It was used to hold the proximal free ends of the graft loops and pull them gently into the posterior laminectomy incision.

### Side-to-side end-to-side grafting neurorrhaphy between the recipient brachial plexus and the distal aspect of the nerve graft conduits

Side-to-side end-to-side grafting neurorrhaphy between the recipient brachial plexus and the distal aspect of the nerve graft conduits is the second issue we have to consider. In a previous clinical study [30], we introduced several end-to-side side-to-side grafting neurorrhaphy techniques. In the intranervous closed loop technique nerve grafts were passed (looped) into slits made into the distal nerve stumps and side grafted to them. Contrary to conventional fascicular epiperineurial neurorrhaphy, this created a stable recipient-graft junction and allowed for an increased contact area between the grafts and the recipient nerves. In that study, we also introduced the principle of single donor to multiple recipient neurotization. Success of that procedure depended upon choosing a donor with high axonal count [14,39,40], on increasing the number of grafts and on increasing the recipient-graft and graft donor contact areas [30,41]. Only through this could several muscles reach motor power greater than Grade 3 without cocontractions.

In the present work, closed looping provided a stable graft recipient junction, which allowed turning the patient again into the lateral position to approach the cervical cord posteriorly; it also allowed retrieving of the proximal ends of the grafts fom the anterior to the posterior field. In C5,6,7, 8T1 avulsions and using the principle of closed loop of end-to-side side-to-side grafting neurorrhaphy [30], one nerve graft was looped through the superior and middle trunks and lateral and posterior cords and another nerve graft was looped through the inferior trunk, medial cord and medial root of median nerve. In C5,6 ruptures C7,8 T1 avulsions, after grafting ruptures, one nerve graft was looped through the middle trunk and posterior cord and another was looped through the inferior trunk, medial cord and medial root of median nerve.

### Side-to-side end-to-side grafting neurorrhaphy between the donor anterior aspect of the cervical cord and the proximal ends of the nerve graft conduits

Side-to-side end-to-side grafting neurorrhaphy between the donor anterior aspect of the cervical cord and the proximal ends of the nerve graft conduits is the third issue we have to address..

After root avulsion from the spinal cord, there is degeneration of sensory and motor axons of spinal motoneurons, loss of synapses, deterioration of local segmental connections, nerve cell death and reactions among non neuronal cells with scar formation [4]. Nevertheless, motoneurons are able to regenerate; new nerve fibers grow along a trajectory consisting of central nervous system (CNS) growth-inhibitory tissue in the spinal cord as well as peripheral nervous system PNS growth-promoting tissue in nerves [4]. Several theories have been advanced to account for the limited regenerative capacity of the central nervous system.: the physical characteristics of the glial scar, inhibitory cell surface or extracellular matrix molecules (such as axon growth-inhibitory proteoglycan NG2 [42]), a lack of suitable guidance channels and substrates, the presence of myelin associated growth-inhibiting molecules, an absence of growth-promoting neurotrophic molecules and the cell intrinsic growth potential motoneurons [43].

Nonetheless, avulsed nerve roots could be reimplanted into the spinal cord [44-51]. It was concluded that central nervous tissue axons might grow through scar tissue that had a persistent defect in the blood-brain barrier [52]. Blood borne cells such as macrophages and T cells invaded the scar, and through their release of interleukins tumor necrosis factor, interferon gamma and prostaglandins contributed to upregulation by astrocytes of neurotrophins and extracellular matrix molecules, such as laminin, and neurotrophins [3]. After ventral root avulsion, mRNAs for receptors or receptor components for neurotrophin-3 (NT-3), ciliary neurotrophic factor (CNTF) and leukemia inhibitory factor (LIF) were strongly downregulated, while receptors for glial cell line-derived neurotrophic factor (GDNF) and laminins were profoundly upregulated [53]. Both laminin-2 (alpha2beta1gamma1) and laminin-8 (alpha4beta1gamma1) were important for axonal regeneration after injury [54]. The production of nerve growth factor by the astrocytes, was shown to attract leptomeningeal cells to participate in the formation of a trabecular scar. These leptomeningeal cells, in turn, were shown to express the low affinity neurotrophin receptor p75 [3].

However, neurons could not elongate across the peripheral (PNS)-central nervous system (CNS) transitional zone. The astrocytic rich central nervous system part of the spinal nerve root prevented regeneration even of nerve fibers from transplanted embryonic ganglion cells. Regeneration of severed nerve fibers into the spinal cord occurred when the transition zone was absent as in the immature animal [55]. Thus to reestablish spinal cord to peripheral nerve connectivity, the transitional region should be deleted and severed ventral or dorsal roots directly implanted into the spinal cord [55,56]. This procedure formed a kind of side neurotization between the donor cord side to the end and side of the recipient nerve graft. Interestingly, the same procedure also seemed to have an attenuating effect on the pain that developed in cases with a combined dorsal root avulsion [56].

Nevertheless, problems in nervous regeneration such as non directional growths and unspecific reinnervation of target organs led to unpredictable sensorimotor activity and conspired against a useful recovery of function [4]. After ventral nerve root implantation, different functional pools of motor neurons were attracted to regrow axons on the implanted root as judged by their position in the ventral horn [3]. However, neurons normally supplying an antagonist muscle, such as the triceps muscle, might participate in the innovation of the biceps muscle, thus leading to cocontractions among antagonistic muscles. Strategies improving the number of motor fibers regenerating into the reimplanted ventral roots and possibly extending regeneration to distal musculature include: the placement of peripheral nerve grafts, the grafting of fetal neurons or of olfactory ensheathing cells [57,58] (neural transplantation), the application of growth factors or Schwann cells to the area of the spinal lesion, the blockade of inhibitory molecules [43], or the genetic modification of peripheral nerve grafts to overexpress outgrowth-promoting proteins [59-62]. However, high levels of neurotrophic factors in the ventral horn might prevent directional growth of axons of a higher number of surviving motoneurons into the implanted root [63].

In a previous clinical study [30], we introduced several end-to-side side-to-side grafting neurorrhaphy techniques. In the long length contact technique, we used both of the cut end and side of contralateral C7 to neurotize the lateral, medial and posterior cords of the brachial plexus simultaneously. Recovery was marked by being associated with cocontractions and by being differential in nature, some muscles recovering better than others, agonists recovering better than antagonists, proximal muscles recovering better than distal muscles. Achieving functional motor power in several muscles without cocontractions depended upon choosing a donor with high axonal count [14], on increasing the number of grafts and on increasing the recipient-graft and graft donor contact areas [30,41].

Current approaches used for direct cord implantation afford little exposure to the cord and intradural nerve roots, thus limiting the area of side neurotization to the cord.

In the present work, the dura was opened posteriorly using a 11-scalpel blade. Its edges were kept open by means of 3/0 prolene sutures. A dural dissector was used to clear the pia mater off the anterior cord from C4 up to C7. The grafts were passed through the dural incision and placed in an end(graft)-to-side(cord) and side(graft)-to-side(cord) fashion for about 4 cms along the anterior cord close to the midline sulcus in a subpial plane. They were held in place by placing them anterior to C4 intradural cervical nerve root proximally and to T1 intradural nerve root distally. These procedures allowed for side neurotization between the donor cord and the nerve grafts along a broad surface area. This became only possible because of the adequate exposure and extensibility provided by the posterior cervical laminectomy.

### The role of direct cord implantation in complete and incomplete avulsions

The role of direct cord implantation in complete and incomplete avulsions are the fourth and fifth issues we have to consider. This was carried out experimentally on monkeys, cats and rats [64-67]. The C5-C8 ventral roots were avulsed in Macaca fascicularis monkeys and reimplanted into the ventrolateral part of the spinal cord either immediately or after a delay of 2 months. There was substantial recovery of function especially after immediate, less so after delayed spinal cord implantation. Cocontractions occurred [64,65]. Clinically, motor function significantly improved after re-implanting avulsed spinal roots directly to the spinal cord [3,8-11,68]. Motor function might be restored throughout the arm, forearm and hand when 1 or 2 avulsed roots were reimplanted into the cord, while the others were intact [9]. Motor function might even be restored in the intrinsic muscles of the hand when surgery was performed in the paediatric age group [10]. However, cocontractions of agonist and antagonist muscle groups were reported to occur clinically. Spontaneous contractions of limb muscles in synchrony with respiration, the "breathing arm" might also ensue [3,8-10,68]. Clinically, pain intensity was significantly correlated with the number of roots avulsed prior to surgery; surgical repairs were associated with pain relief. Sensory recovery to thermal stimuli was observed, mainly in the C5 dermatome. Allodynia to mechanical and thermal stimuli was observed in the border zone of affected and unaffected dermatomes. Pain and sensations referred to the original source of afferents as well as "wrong-way" referred sensations (e.g. down the affected arm while shaving or drinking cold fluids) might occur [69]. Early repair was more effective than delayed repair in the relief from pain and there was a strong correlation between functional recovery and relief from pain [70].

In the current series, Cases 1 and 2 were complete avulsions in patients, in whom surgery was performed within 1 year after injury. The biceps and anterior, lateral and posterior deltoid, the supraspinatus, the subscapularis, pectoral and clavicular heads of pectoralis major, latissimus dorsi, improved from Grade 0 to Grade 4. The infraspinatus, triceps and pronator teres improved from Grade 0 to Grade 3. Thus there was nearly complete improvement in shoulder and elbow functions; improvement extended even into the forearm. Cocontractions were recorded.

Because of weak shoulder external rotation, Cases 1 and 2 achieved a Narakas and a Waikakul score of good; they remained with a Raimondi score of 0..

Cases 3 and 4 were C5,6 ruptures C7,8T1 avulsions. The biceps and anterior deltoid improved from Grade0 to Grade5; the lateral and posterior deltoid, the supra- and infraspinatus, the subscapularis, pectoral and clavicular heads of pectoralis major, latissimus dorsi, triceps improved from Grade 0 to Grade 4. The pronator teres, extensor carpi ulnaris, flexor digitorum profundus and flexor pollicis longus improved from Grade 0 to Grade 3. The flexor digitorum superficialis improved from Grade 0 to Grade 2. Thus there was almost complete improvement in shoulder and elbow functions; improvement extended even into the forearm and hand. No cocontractions were recorded. Both cases achieved a Narakas score of excellent, a Waikakul score of excellent. and improved from a Raimondi score of 0 to a score of 3. Improvement of forearm and hand function when 1 or 2 avulsed roots were reimplanted into the cord, while the others were intact conformed with other reports in the literature [9]. Interestingly, however, no cocontractions were recorded. Of equal interest was the disappearance of pain after cord reimplantation in these cases, but not in total avulsions [70].

In Case 5, the biceps and anterior deltoid improved from Grade0 to Grade5; the lateral and posterior deltoid, the supraspinatus, the subscapularis, pectoral and clavicular heads of pectoralis major, latissimus dorsi, triceps improved from Grade 0 to Grade 4. The infraspinatus, pronator teres, extensor carpi ulnaris, extensor carpi radialis longus and brevis, flexor carpi ulnaris, flexor carpi radialis, thumb and finger extensors, flexor digitorum profundus and superficialis, flexor pollicis longus improved from Grade 0 to Grade 3. The intrinsic muscles of the hand improved from Grade 0 to Grade 2. Cocontractions occurred between the lateral deltoid, biceps and finger extensors on active shoulder abduction. Case 5 achieved a Narakas score of good, a Grade3 Gilbert shoulder score, a Gilbert elbow score of good and a Raimondi score of 0 to a score of 4. These findings were in accordance with those reported in the literature of restoration of hand function in the paediatric age group [10]. Contrary to adult cases, cocontractions persisted, however.

### Shortcomings of the technique

Sixth, there are still shortcomings of the technique. As mentioned before, direct cord implantation is a kind of single donor to multiple recipient neurotization. Success of that procedure depends upon choosing a donor with high axonal count (the spinal cord), on increasing the number of grafts and on increasing the recipient-graft and graft donor contact areas. Only through this could several muscles reach motor power greater than Grade 3 without cocontractions. We managed to increase the recipient-graft contact area by using closed loop grafting neurorrhaphy [30]. The graft donor contact area was increased by placing the grafts in an end(graft)-to-side(cord) and side(graft)-to-side(cord) fashion for about 4 cms along the anterior cord close to the midline sulcus in a subpial plane.

Increasing the number of grafts, however, is limited by the number of sensory nerves that could be used as grafts. This incited scientists to develop synthetic nerve grafts. Actually, a natural nerve graft should be considered as a complex of proportionate amounts of Schwann cells, neurotrophic factors, cell adhesion molecules and neurite-outgrowth-promoting factors (such as laminin), all four of which are essential to axonal regeneration [71]. Simply applying high levels of neurotrophic factors alone to nerve roots directly implanted into the ventral horn without adding proportionate amounts of cell adhesion molecules and neurite-outgrowth promoting factors (laminin) might explain the poor result obtained by other authors [63,72]. The adequate exposure provided by our approach allows not only for placing many side grafts along an extensive donor recipient area, but for associating them with an expandable amount of synthetic grafts as well.

### Limitations of this study

Seventh, we should point out the limitations of this study. We reported cocontractions and Grade4 shoulder improvement in total avulsions; absence of cocontractions, Grade4-5 shoulder improvement and extension of improvement into the forearm in C5,6ruptures, C7,8T1 avulsions. These are relatively good results in relatively poor situations. We attributed this to enhanced side neurotization due the extensive contact areas between the cord (high axonal load donor) and the grafts proximally and between the grafts and brachial plexus trunks, divisions and cords distally. This should be weighed, however, against other aspects: spontaneous recovery despite MRI appearance of avulsions, fallacies in intraoperative determining of avulsions (wrong diagnosis, wrong level); small sample size; no controls rule out superiority of this technique versus other direct cord reimplantation techniques or other neurotization procedures; intra- and interobserver variability in testing muscle power and cocontractions.

Nevertheless, direct cord implantation is now an established procedure. It is therefore hoped, the single stage combined anterior (first) posterior (second) approach approach might stimulate brachial plexus surgeons to go more for direct cord implantation, whether they use side grafting, fibrin glue, end to end grafting or any other established neurorrhaphy techniques.

## Conclusion

Through providing proper exposure to the brachial plexus and to the cervical cord, the single stage combined anterior (first) posterior (second) approach might stimulate brachial plexus surgeons to go more for direct cord implantation. In this study, it allowed for placing side grafts along an extensive donor recipient area by end-to-side side-to-side grafting neurorrhaphy and thus improved results.

## Consent

Written informed consent was obtained from the patients for publication of the accompanying images.

## Competing interests

The authors declare that they have no competing interests.

## Authors' contributions

All authors were involved in the conception and design of the study. All authors read and approved the final manuscript. SMA wrote the rough draft. Brachial plexus exploration was carried out by SMA, ANM, RERE. Cervical laminectomy was carried out by SMA and AMK. Extraction of the nerve grafts was performed by AME, AMSA

## Supplementary Material

Additional file 1 **The pre- and postoperative motor power grades of the individual muscles in each patient**. Table 2 representing the pre- and postoperative motor power grades of the individual muscles in each patient Click here for file
