# Supplementary material for: Direct cord implantation in brachial plexus avulsions: revised technique using a single stage combined anterior (first) posterior (second) approach and end-to-side side-to-side grafting neurorrhaphy
Source: J Brachial Plex Peripher Nerve Inj. 2009 Jun 19;4:8. doi: 10.1186/1749-7221-4-8 (PMC2711067; doi:10.1186/1749-7221-4-8)
Supplement: Additional file 1 — The pre- and postoperative motor power grades of the individual muscles in each patient. Table 2 representing the pre- and postoperative motor power grades of the individual muscles in each patient [file 1749-7221-4-8-S1.doc]

|  |  | |  | | | | | |  | | | | | |  | | | |  | |  | |  | |  | |  | | | |  | | | |  | |  | | | |  | | | | | | | |  | | | |
| --- | --- | --- | --- | --- | --- | --- | --- | --- | --- | --- | --- | --- | --- | --- | --- | --- | --- | --- | --- | --- | --- | --- | --- | --- | --- | --- | --- | --- | --- | --- | --- | --- | --- | --- | --- | --- | --- | --- | --- | --- | --- | --- | --- | --- | --- | --- | --- | --- | --- | --- | --- | --- |
| Pt | Bi=  ceps | | Deltoid | | | | | | Rotator cuff ms. | | | | | | Pectoralis  major | | | | Lat. dorsi | | Triceps | | Fore=  arm pron. | | Fore=  arm  sup. | | Wrist  extensors  (extrs.) | | | | Wrist flexors | | | | Finger extrs. | | Finger flexors | | | | Thumb | | | | | | | | Intrinsic  muscles | | | |
|  |  | | ant | | lat | | post | | Supra=  spin=  atus | | Infra=  spin=  atus | | Sub=  scap=  ularis | | Clav. head | | Pect.  head | |  | |  | | Pron.  teres | | Supi=  nator | | Ulnar (ECU) | | Radial (ECRL  &br.) | | Ulnar (FCU) | | Radial (FCR) | |  | | FDS to Ds2-5 | | FDP to Ds2-5 | | FPL | | EPL | | EPbr. | | Abd. Poll. | | Suppl. by ulnar n. | | Suppl. by median n. | |
|  | C5,6 | | C5,6 | | C5,6 | | C5,6 | | C(4),5,  6 | | C(4),5,  6 | | C5,6, (7) | | C5,6 | | C7,8  T1 | |  | |  | | C6,7 | | C5,6 | | C7,8 | | C6,7-  C7,8 | | C7,8 | | C6,7 | | C7,8 | | C7,8T1 | | C8T1 | | C8T1 | | C7,8 | | C7,8 | | C7,8 | | C8T1 | | C7,8 | |
|  | pre/  post | | pre/  post | | pre/  post | | pre/  post | | pre/  post | | pre/  post | | pre/  post | | pre/  post | | pre/  post | | pre/  post | | pre/  post | | pre/  post | | pre/  post | | pre/  post | | pre/  post | | pre/  post | | pre/  post | | pre/  post | | pre/  post | | pre/  post | | pre/  post | | pre/  post | | pre/  post | | pre/  post | | pre/  post | | pre/  post | |
|  |  | |  | |  | |  | |  | |  | |  | |  | |  | |  | |  | |  | |  | |  | |  | |  | |  | |  | |  | |  | |  | |  | |  | |  | |  | |  | |
|  |  |  |  |  |  |  |  |  |  |  |  |  |  |  |  |  |  |  |  |  |  |  |  |  |  |  |  |  |  |  |  |  |  |  |  |  |  |  |  |  |  |  |  |  |  |  |  |  |  |  |  |  |
| 1 | 0 | 4 | 0 | 4 | 0 | 4 | 0 | 4 | 0 | 4 | 0 | 3 | 0 | 4 | 0 | 4 | 0 | 4 | 0 | 4 | 0 | 3 | 0 | 3 | 0 | 0 | 0 | 0 | 0 | 0 | 0 | 0 | 0 | 0 | 0 | 0 | 0 | 0 | 0 | 0 | 0 | 0 | 0 | 0 | 0 | 0 | 0 | 0 | 0 | 0 | 0 | 0 |
| 2 | 0 | 4 | 0 | 4 | 0 | 4 | 0 | 4 | 0 | 4 | 0 | 3 | 0 | 4 | 0 | 4 | 0 | 4 | 0 | 4 | 0 | 3 | 0 | 3 | 0 | 0 | 0 | 0 | 0 | 0 | 0 | 0 | 0 | 0 | 0 | 0 | 0 | 0 | 0 | 0 | 0 | 0 | 0 | 0 | 0 | 0 | 0 | 0 | 0 | 0 | 0 | 0 |
| 3 | 0 | 5 | 0 | 5 | 0 | 4 | 0 | 4 | 0 | 4 | 0 | 4 | 0 | 4 | 0 | 4 | 0 | 4 | 0 | 4 | 0 | 4 | 0 | 3 | 0 | 0 | 0 | 3 | 0 | 0 | 0 | 0 | 0 | 0 | 0 | 0 | 0 | 2 | 0 | 3 | 0 | 3 | 0 | 0 | 0 | 0 | 0 | 0 | 0 | 0 | 0 | 0 |
| 4 | 0 | 5 | 0 | 5 | 0 | 4 | 0 | 4 | 0 | 4 | 0 | 4 | 0 | 4 | 0 | 4 | 0 | 4 | 0 | 4 | 0 | 4 | 0 | 3 | 0 | 0 | 0 | 3 | 0 | 0 | 0 | 0 | 0 | 0 | 0 | 0 | 0 | 2 | 0 | 3 | 0 | 3 | 0 | 0 | 0 | 0 | 0 | 0 | 0 | 0 | 0 | 0 |
| 5 | 0 | 5 | 0 | 5 | 0 | 4 | 0 | 4 | 0 | 4 | 0 | 3 | 0 | 4 | 0 | 4 | 0 | 4 | 0 | 4 | 0 | 4 | 0 | 3 | 0 | 0 | 0 | 3 | 0 | 3 | 0 | 3 | 0 | 3 | 0 | 3 | 0 | 3 | 0 | 3 | 0 | 3 | 0 | 3 | 0 | 3 | 0 | 3 | 0 | 2 | 0 | 2 |
|  |  |  |  |  |  |  |  |  |  |  |  |  |  |  |  |  |  |  |  |  |  |  |  |  |  |  |  |  |  |  |  |  |  |  |  |  |  |  |  |  |  |  |  |  |  |  |  |  |  |  |  |  |
|  |  |  |  |  |  |  |  |  |  |  |  |  |  |  |  |  |  |  |  |  |  |  |  |  |  |  |  |  |  |  |  |  |  |  |  |  |  |  |  |  |  |  |  |  |  |  |  |  |  |  |  |  |
